# The influence of smoking and other risk factors on the outcome after radiochemotherapy for anal cancer

**DOI:** 10.1186/1748-717X-2-30

**Published:** 2007-08-21

**Authors:** Sabine Kathrin Mai, Grit Welzel, Verena Haegele, Frederik Wenz

**Affiliations:** 1 Department of Radiation Oncology of the University Medical Center Mannheim, Germany

## Abstract

**Background:**

Smoking is an important risk factor for the development of cancer. Smoking during radiochemotherapy therapy may have a negative influence on prognosis. We evaluated the effect of smoking during radiochemotherapy on the outcome for patients with anal cancer.

**Methods:**

Sixty-eight patients (34 smokers, 34 non-smokers) treated by radiochemotherapy for anal cancer were analysed. The effect of smoking during radiochemotherapy and other risk factors (gender, T- and N category, tumor site, dose, therapy protocol) on disease-specific survival (DSS), local control (LC) and colostomy free survival (CFS) was evaluated.

**Results:**

There was a significant difference in age and male:female ratio between the two groups. With a median follow up of 22 months (max. 119) DSS, LC, and CFS were 88%, 84% and 84%. A significant difference in local control between smokers (S) and non-smokers (NS) was found (S 74% vs. NS 94%, p = .03). For DSS and CFS a difference in terms of outcome between smokers and non-smokers was seen (DSS: S 82% vs. NS 96%, p = .19, CFS: S 75% vs. 91%, p = .15), which did not reach statistical significance. In multivariate analyses only gender had a significant association with LC and T category with CFS. The other risk factors did not reach statistical significance.

**Conclusion:**

Even though our evaluation reached statistical significance only in univariate analysis, we suggest, that the role of smoking during radiochemotherapy for anal cancer should not be ignored. The potential negative effect on prognosis should be explained to patients before therapy.

## Background

Smoking is one of the most important risk factors for the development of several cancers, especially squamous cell carcinoma [1]. In addition, smokers often have a worse prognosis than non-smokers undergoing anti tumor therapy [2-5]. On the one hand smokers often present with more advanced tumor stages on the other hand smoking especially during therapy seems to have a negative influence on the efficacy of therapy. Browman et al. showed, that patients with head and neck cancer, who smoked during radiochemotherapy had significantly lower survival rates than patients who stopped smoking [6].

Several studies revealed that beside HIV and HPV infection smoking is one of the most important risk factors for anal cancer, especially in combination with virus infection. The risk of developing anal cancer is increased with the combination of one of these risk factors with smoking [7-11].

Combined radiochemotherapy is the gold-standard in treatment of anal cancer since Nigro et al reported their data [12]. Local control rates of 60 – 90% over all stages are achievable with sphincter preservation in about 65% of the cases. There are some known factors influencing the prognosis after combined radiochemotherapy for anal cancer. Higher tumor stage and regional nodal involvement are associated with an inferior prognosis. Also tumor site in the anal canal seems to be associated with unfavourable prognosis, but some authors found tumor site at the anal margin as independent significant prognostic factor for overall survival [13]. Several authors suggest that the prognosis of female patients is superior to the prognosis of male patients [14,15]. However, so far no data about the influence of smoking on the prognosis of anal carcinoma treated with primary combined radiochemotherapy have been published to our knowledge. The aim of this retrospective analysis was to evaluate the correlation between smoking behaviour and other risk factors and the outcome in patients treated with radiochemotherapy for anal cancer in a single center.

## Methods

Between 1990 and 2006 a total of 90 patients were treated with combined radiochemotherapy for anal cancer. Data about smoking behaviour before and during therapy were available from 73 patients. Five patients were lost to follow up. Therefore 68 patients were included in this evaluation. Thirty-four patients were non-smokers and 34 current smokers. Eight patients among the non-smokers had stopped smoking more than 1 year before therapy. One patient in the smoking group was HIV positive. Three non-smokers and six smokers had complete tumor resection before therapy (2 APR, one in each group, and 7 local excision (2 non smokers, 5 smokers)) and were treated in adjuvant intention.

Patients' characteristics are displayed in Table 1.

**Table 1:** Patients' characteristics

	Smoker	Non Smoker	
n	34	34	
mean age (range)	55 (34 – 77)	62 (38–86)	p = 0.21
male:female	18:16	7:27	p = 0.006
Localisation			ns
Anal canal	19	27	
Anal verge	10	2	
Overlapping	5	5	
T-Stage			ns
T1	3	7	
T2	19	19	
T3	10	3	
T4	2	5	
N-Stage			ns
N 0	25	22	
N +	9	12	
Therapy Protocol			ns
Cummings	3	3	
EORTC	1	3	
RTOG	30	28	

The influence of smoking on the actuarial disease specific survival (DSS), local control (LC), and colostomy-free survival (CFS) at 5 years was calculated. In addition the influence of other risk factors like gender, tumor site, tumor- and nodal stage, radiation dose and therapy protocol on these end points was evaluated.

### Therapy

All patients were treated with combined radiochemotherapy according to 3 different protocols (see Table 2). Treatment was performed according to the Cummings protocol until 1997, to the EORTC protocol from 1997 to 1999 and to the RTOG protocol since 1999. In each protocol chemotherapy consists of a combination of Mitomycin C bolus and 5FU continuous infusion. The protocol published by Cummings et al. provided a total dose of 50 Gy with a split course of 4 weeks, the EORTC protocol a total dose of 59,4 Gy with a split course of 2 weeks and the RTOG protocol a total dose of 50–54 Gy dependent on the tumor stage without split.

**Table 2:** Therapy protocols

**Protocol**	**TD (Gy)**	**SD (Gy)**	**Chemotherapy**	**Split**
**Cummings**	48 – 50	1.8 – 2.5	MMC 10 mg/m2 d1	
			5FU 1000 mg/m2/24 h d1–5, 2 cycles	4 wks
**EORTC**	59,4	1.8	MMC 10 mg/m2 d1	
			5FU 200 mg/m2/24 h d1–26 + d1–13,	2 wks
**RTOG**	50–54	1.8 – 2	MMC 10 mg/m2 d1 + 29	
			5FU 1000 mg/m2/24 h d1–4 + 29–32	-

### Follow up

First follow up was performed 6 weeks after the end of therapy and then every 3 months for the first 2 years and then every 6 months for 5 years. After 5 years annual examinations were recommended. The follow up examinations included physical examination, rectoscopy and anorectal ultrasound and CT scan of the abdomen and pelvis. A chest x-ray or CT-scan of the lung was performed every 6 – 12 months. Local failure was defined as tumor persistence within 3 months after therapy or histologically verified recurrence beyond 3 months after therapy.

### Statistical analysis

SPSS software, version 14.0.1 (SPSS Inc., Chicago, IL, USA) was used for statistical analysis. Kaplan-Meier survival curves were generated and compared using the log-rank test. Prognostic factors found to be significantly associated with survival on univariate analysis were entered into a multivariate Cox model using the stepwise backward procedure. For all analyses a two-sided P-value of <0.05 was considered statistically significant.

## Results

Sixty-eight patients with complete data were eligible for evaluation. Thirty-four patients were smokers and 34 patients non-smokers. Median follow up of the whole group was 22 months (range: 2 – 119). Related to smoking status there was no relevant difference in follow up (smokers 24 months (min 2, max 119), non-smokers 22 months (min 2, max 111). Mean age of the smokers was 55 years (range: 34–77) and of the non-smokers 62 years (38 – 86). The male:female ratio was significantly shifted to males among the smokers (p = .006). Respective to tumor localisation, tumor and nodal stage there was no relevant difference between the two groups. Also there was no difference in therapy protocol. Most patients in both groups were treated according to the RTOG protocol. (see Table 2).

### Therapy

At the beginning of therapy 32 smokers and 31 non-smokers were colostomy-free (smokers: 1 abdomino perineal resection for anal cancer, 1 protective colostomy for anal abscess, non-smokers 1 abdominoperineal resection for anal cancer and 2 protective colostomies for anal abscess or fistula). All patients except one non-smoker received the planned total dose of radiotherapy. In this case radiochemotherapy was stopped after first cycle of chemotherapy at a dose of 43 Gy because of toxicity and old age of the patient. The intensity of chemotherapy was reduced in 1 smoker and 6 non-smokers due to hematologic side effects. Most patients with therapy reductions were treated according to the EORTC protocol.

Mean dose of radiotherapy was 51.9 Gy (43 – 59,4 Gy). Eight patients received a total dose of less than 50 Gy. Four patients had a complete tumor resection (2 APR and 2 excision) and were treated in adjuvant intention with a dose of 45 Gy, one patient stopped therapy (43 Gy, see above) and in 2 patients dose prescription was 49 Gy.

#### Disease specific survival (DSS)

**Figure 1:**
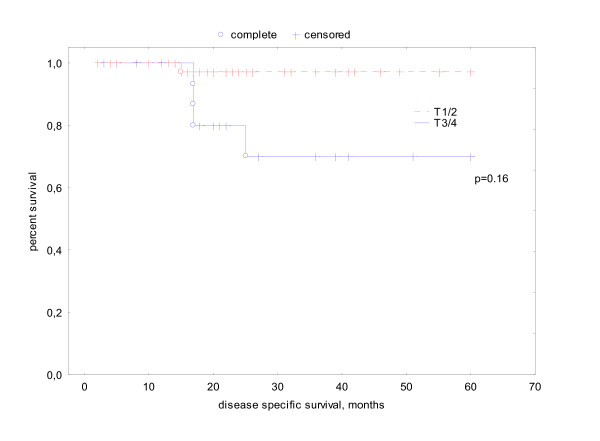
actuarial disease specific survival related T-stage.

**Figure 2:**
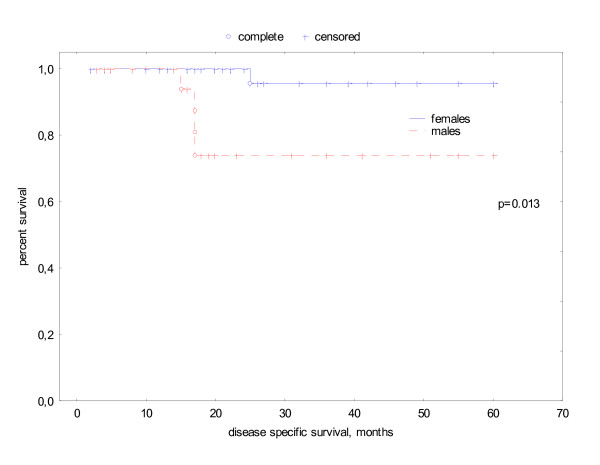
actuarial disease specific survival related to gender.

Sixty-one patients were alive at the date of evaluation. 5 patients died disease related, 2 patients intercurrently resulting in an actuarial DSS of 88%. The DSS of smokers versus non-smokers was 82% vs. 96% (p = 0.19). Disease specific survival had a significant association with tumor stage (T1/2 97% vs T3/4 70% p = .016, log rank test) and gender (male 74% vs. female 96% p = .013) (see Fig. 1, 2). Because of the small number of tumor related deaths multivariate analysis was not meaningful.

#### Local Control (LC)

**Figure 3:**
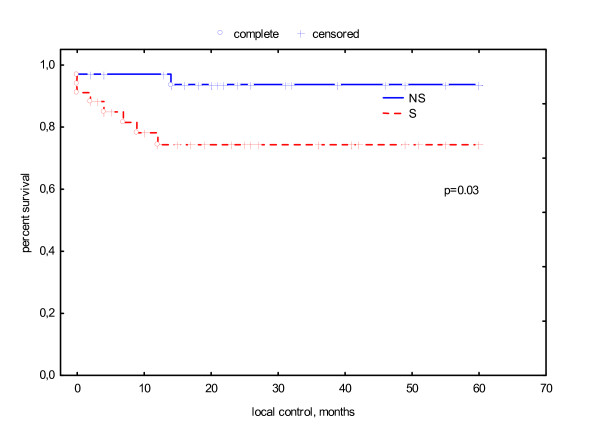
actuarial local control related to smoking status.

**Figure 4:**
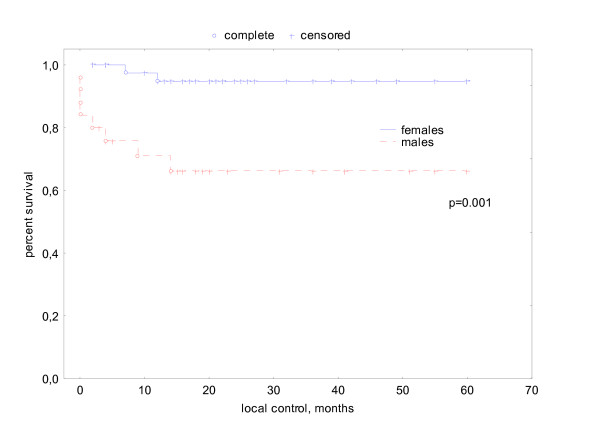
actuarial local control related to gender.

Ten patients suffered from recurrent disease. Two of these patients had an anal carcinoma related to a giant condyloma Buschke-Löwenstein, 2 patients had anal cancer in recurrent anal fistulas and abscesses because of chronic inflammatory bowel disease, 1 patient had synchronic renal cancer, 1 patient was permanently immunosuppressed because of Myasthenia gravis and one patient was HIV positive. Tumor stages in these 10 patients were T1 n = 1, T2 n = 4, T3 n = 4, T4 n = 1 and N+ n = 4. All recurrences appeared within the first 2 years after therapy. This results in an actuarial local control rate at 5 years of 84%. Local control had a significant association with smoking (S 74% versus NS 94%, p = .03, log rank test) and gender (male 66% vs. female 95%, p = .001, log rank test) (see Fig. 3, 4). In multivariate analysis gender was the only variable significantly determining local control. (see Table 3).

**Table 3:** Results of the Cox regression analysis (stepwise backward procedure)

**Variable**	**Score**	**Coeffizient**	**SE**	**HR**	**95% CI**	**P**
(a) Step 1						
Gender						
Male (n = 25)	1	1.81	0.82	6.08	1.23–30.15	0.027
Female (n = 43)	0					
Smoking						
Yes (n = 34)	1	1.01	0.82	2.75	0.55–13.64	0.217
No (n = 34)	0					
(b) Step 2						
Gender						
Male (n = 25)	1	2.09	0.79	8.12	1.72–38.32	0.008
Female (n = 43)	0					

Abbreviations: SE – Standard Error, HR – Hazard Ratio, CI-Confidence Intervall

When local control calculated separately for males and females, there was a difference between smoking and non smoking females for local control with a strong tendency towards significance (LC: NS 100% vs. S 87,5%, p = .054). Among the males there was also a difference, but not reaching statistical significance (LC: NS 71,4% vs. 66,7%, p = 0.69).

#### Colostomy free survival (CFS)

**Figure 5:**
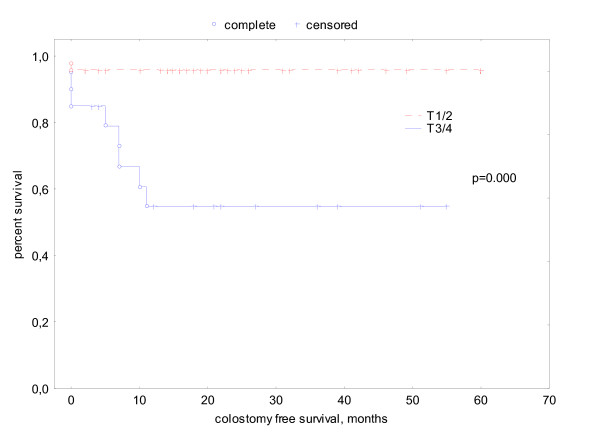
actuarial colostomy free survival related to t-stage.

63 patients were colostomy free at the beginning of therapy. The actuarial colostomy free survival at 5 years was 84%. All colostomies were performed because of persistent or recurrent disease, none was done for insufficient sphincter function due to toxicity. Two patients had a salvage abdomino-perineal resection and 3 patients palliative colostomy because of massive sphincter infiltration. CFS of smokers versus non-smokers was 75% vs. 91% (p = 0.15). In univariate log rank tests, T category was the only significant prognostic factor for colostomy free survival. Colostomy free survival in patients with tumor stage T1/2 was 96% versus 55% in patients with tumor stage T3/4 (p = .000). (see Fig. 5) The other prognostic factors showed no significant influence on colostomy free survival.

### Other risk factors

The remaining risk factors like N stage, tumor localisation, radiation dose and therapy protocol did not reach statistical significance.

## Discussion

While smoking as a risk factor for the development of anal cancer is well known, no data about the influence of smoking during combined radiotherapy on the outcome after therapy exist as of now. The exact role of smoking in the etiology of anal cancer is still somewhat unclear. One hypothesis is, that nicotine acts as a promoter for malignant transformation in cells with HPV-DNA [16]. Phillips et al. found elevated levels of DNA adducts in anal epithelium of smokers [17]. Other potential aspects are the inhibition of apoptosis or immunosupression caused by smoking [18-22]. Smoking also is associated with higher levels of carboxyhemoglobin resulting in tissue hypoxia. Especially the inhibition of apoptosis and tissue hypoxia may have an influence on the efficacy of radiochemotherapy.

Browman et al. studied this effect in patients receiving radio(chemo)therapy for head and neck cancer. They found that smoking was an independent prognostic factor for survival with a relative risk of 2.3. Also the smoking history was identified as additional factor influencing survival [6]. In the follow up study from 2002 this effect showed not longer statistical significance but in univariate analysis there was still a survival difference favouring light smokers vs. heavy smokers [23]. Several other studies also found a significant effect on outcome for patients smoking during radio/chemotherapy for non small cell and small cell lung cancer [24,25].

As in the follow up study from Browman, in our study smoking as prognostic factor did not reach statistical significance in multivariate analysis for the limit of the retrospective study. In log rank tests smokers had significantly more local failures than non smokers and also for disease specific survival and colostomy free survival there was a trend toward better outcome for non smokers. Overall the number of tumor related deaths and colostomies in our study was small because of the good prognosis of anal cancer after combined radiochemotherapy. According to the retrospective character of our evaluation we only had information, whether the patients smoked or not and when they stopped smoking. No exact quantitative data about the number of smoked cigarettes or pack years were available. One may speculate whether with a larger number of patients and more detailed quantitative information smoking might turn out as an independent prognostic factor in multivariate analysis.

We found gender as independent prognostic factor for local control and disease free survival in multivariate analysis. There was no difference in tumor- or therapy related factors between males and females, but the male:female ratio was shifted towards a higher number of males among the smokers. The role of gender in the prognosis of anal cancer remains still unclear. Some series and our data suggest, that men suffering from anal cancer have a poorer prognosis than woman [14,26], whereas others did not [27-29].

When local control of males and females was calculated separately, we saw a difference among female smokers and non smokers with a strong tendency to better prognosis of non smoking females. None of the female non smokers suffered from local recurrence. Also among males there was a difference not reaching statistical significance, caused by the small number of male patients at all and non smoking males. We think both, gender and smoking have influence on local control, although we could not demonstrate significance on the basis of our data. One may speculate that the imbalanced distribution of gender in both groups diminish our ability to detect the potential independent effect of smoking during radiochemotherapy for anal cancer. This should be evaluated in future studies.

T stage and tumor size are well known as prognostic factors for anal cancer [30]. In our analysis T stage had a significant association with DSS and CFS, but not with local control, which may be explained by the small number of recurrences among our patients.

The other evaluated risk factors, in particular N category and tumor site showed no significant prognostic effect. Especially N category is often addressed as a prognostic factor for survival [15,27]. However, other studies also found no significant effect of nodal status [28,31] which may be caused by the heterogeneous treatment modalities in the respective studies. Also we found no difference between the three treatment protocols. On the other hand only 10 patients were treated outside the RTOG protocol and the differences between the three protocols in dose and chemotherapy are only marginal.

## Conclusion

In conclusion, even though our evaluation showing a negative influence of smoking on outcome reached statistical significance only in univariate analysis, we suggest, that the role of smoking during radiochemotherapy for anal cancer should not be ignored. Therefore the negative effect that smoking might have on their prognosis should be explained to patients before therapy.

## Abbreviations

DSS- Disease specific survival

LC- Local control

CFS- Colostomy free survival

S- Smoker

NS- Non-smoker

## Competing interests

The author(s) declare that they have no competing interests.

## Authors' contributions

SM participated in the conception and design of the study, SM, GW and VH collected the data. SM and GW performed the statistical evaluation. FW participated in design of the study and revised the manuscript.
